# Evaluation of In-Vitro Studies of the Shalmali Extract on Human Endometrial Stromal Cells

**DOI:** 10.7759/cureus.60699

**Published:** 2024-05-20

**Authors:** Anandhan P, Muttevi Hayagreva Kumar, Saad H Elshafey, Janaki C S, Sumathi Jones, Dhastagir Sheriff, Akshara Pramod Roy, Gopi Ayyaswamy, Balaji TK, Prabhu K

**Affiliations:** 1 General Medicine, Sree Balaji Medical College and Hospital, Chennai, IND; 2 Anatomy, St. Joseph College of Health and Allied Sciences, Dar es Salaam, TZA; 3 Anatomy, Northern Border University, Arar, SAU; 4 Anatomy, Bhaarath Medical College and Hospital, Chennai, IND; 5 Pharmacology and Therapeutics, Sree Balaji Dental College and Hospital, Chennai, IND; 6 Biochemistry, Anna Medical College, Montagne Blanche, MUS; 7 Anatomy, Sree Balaji Medical College and Hospital, Chennai, IND; 8 Ear, Nose, and Throat (ENT), Sree Balaji Medical College and Hospital, Chennai, IND; 9 Anatomy, Chettinad Hospital and Research Institute, Chettinad Academy of Research and Education, Chennai, IND

**Keywords:** t hesc cells, phytotherapy, uterine disorders, gene expression analysis, shalmali extract, abnormal uterine bleeding

## Abstract

The utilization of herbal formulations for the management of reproductive tract disorders has been a longstanding practice in traditional medicine. In this study, we investigated the efficacy of a herbal extract, Shalmali (Bombax ceiba), in addressing uterine bleeding, a common concern in gynecological health. Through gene expression analysis, this study examined the impact of Shalmali extract on key genes associated with uterine bleeding, namely ESR1, CD56, and SDF-1, in the human endometrial stromal cell line (T HESC). Our findings revealed a dose-dependent decrease in ESR1 and CD56 gene expression levels following treatment with Shalmali extract, suggesting its potential to modulate hormonal and cellular processes involved in uterine bleeding. Notably, an increase in SDF-1 gene expression was observed, indicating a possible role of Shalmali extract in promoting tissue repair and regeneration. Comparison with the standard drug tranexamic acid demonstrated similar effects on gene expression levels, further validating the therapeutic potential of Shalmali extract. Agarose gel electrophoresis images supported these findings, showing reduced gene expression in cells treated with Shalmali extract comparable to those treated with tranexamic acid. These results underscore the promising efficacy of Shalmali extract as a natural alternative for managing uterine bleeding, potentially offering a safe and effective treatment option for individuals seeking traditional remedies for gynecological concerns. Further research is warranted to elucidate the underlying mechanisms of action and assess the long-term safety and efficacy of Shalmali extract in clinical settings.

## Introduction

Between menarche and menopause, women of reproductive age go through monthly cycles of hormone-regulated endometrial proliferation, differentiation, and shedding. The process of endometrial decidualization mainly consists of prime stages that include the transformation of endometrial stromal cells (ESCs) into a decidualized state, the recruitment of immune cells, the reconstruction of blood vessels, and the abundant production of molecules. These steps collectively play a crucial role in preparing the uterus to support embryo implantation. Variations of the endometrium, comprising both biochemical and morphological adaptations, are arbitrated throughout the menstrual cycle through estrogen and progesterone. During the follicular phase, heightened levels of preovulatory estrogen stimulate the thickening of the endometrium. Estrogen, acting through its receptors, prompts the normal proliferation of endometrial epithelial and stromal cells. However, excessive estrogen can potentially trigger endometrial tumorigenesis. Conversely, progesterone acts as a natural suppressor of endometrial tumors, counteracting the proliferative effects of estrogen. It accomplishes this by inhibiting inflammation, halting the cell cycle, and encouraging differentiation and apoptosis of glandular epithelial cells. Any disruption in the balance between progesterone and estrogen, whether due to prolonged estrogenic stimulation or hindered progesterone activity, can lead to excessive growth of the endometrium. Hypoxia-thrombin interactions may exacerbate the conditions by increasing the expression of vascular endothelial growth factor (VEGF) and IL-8 by stromal cells. Additionally, thrombin, VEGF, and IL-8 collectively enhance angiogenesis in an environment characterized by reactive oxygen species (ROS)-induced activation of endothelial cells. This heightened angiogenesis leads to increased vessel fragility and contributes to abnormal uterine bleeding [1].

Estradiol, the biologically active variant of estrogen, operates via nuclear estrogen receptors to regulate the growth of endometrial tissue. These nuclear receptors ESR1 (estrogen receptor 1) and ESR2,( estrogen receptor 2), arise from distinct genes yet share a remarkable 96% similarity in their DNA binding domains. ESR1 and ESR2 exist in the endometrium, whereas ESR1 takes precedence, and governs the proliferation driven by estrogen. ESR1, binding results in the complex functioning as the transcription factor, attaching to the promoters of estrogen-responsive genes, such as PGR, thereby triggering their activation. ESR1 plays a pivotal role in overseeing the growth of endometrial tissue and is chiefly responsible for mediating estrogen's impacts within the endometrium. Estrogens are essential mediators of endothelial function and can trigger and secrete nitric oxide, endothelial factor, and prostacyclin, and reduce the secretion of vasoconstricting factors (endothelin I & angiotensin II) [2, 3]. Among the estrogen receptors, the ERα receptor has the most important role in the signal transmission of the endothelium [4]. Therefore, any modification in the expression of the gene ESR1 coding for the ERα receptor would alter the endothelial signal transmission and would therefore, additionally enhance the risk of abnormal uterine bleeding in girls with low birth weight.

Earlier studies consistently reported diminished ESR1 levels in both endometriomas and cultured endometriotic stromal cells acquired from ovarian cysts. In the case of ovarian endometriomas, ESR1 mRNA expression is roughly sevenfold lower in comparison to normal endometrial stromal cells. Research utilizing a mouse model has emphasized ESR1's critical role within the uterus and neuroendocrine system, with female mice lacking ESR1 facing infertility due to impaired ovarian and uterine function. Furthermore, aberrant ESR1 expression has been noted in individuals with endometriosis, where ESR1 transcript levels are reduced in women diagnosed with endometriosis when contrasted with those in the control group.

Stromal cell-derived factor 1 (SDF-1) is a growth-stimulating factor that interacts with vascular endothelial growth factors and has a role in influencing the survival and movement of vascular endothelial cells. It also plays a part in altering gene expression. In the context of the human endometrium, SDF-1 appears to have significant roles, especially concerning endometrial growth and the successful implantation of embryos. Reports have suggested that certain blood vessels within the endometrium originate from bone marrow-derived endothelial progenitor cells (EPCs). SDF-1 might be involved in guiding these EPCs to the endometrium, which aligns with the observation that SDF-1 levels in the bloodstream significantly increase during the proliferative phase of the menstrual cycle. Postnatal angiogenesis, the process of forming new blood vessels, occurs in response to disruptions in endothelial integrity. Changes in the integrity of endothelial cells stimulate their proliferation into the surrounding tissue, leading to the re-establishment of the endothelial layer and the formation of neointima, especially following vascular lesions. The levels of SDF-1 in the blood of individuals with heavy menstrual bleeding (HMB) were consistently lower. SDF-1 plays a vital role in regulating vascular remodeling in the endometrium during the menstrual cycle, particularly in response to heavy menstrual bleeding.

The latest study on analyzing the leukocyte population of the functionalis layer of the normal endometrium and abnormal uterine bleeding (AUB)-affected endometrium across the phase of the menstrual cycle observed that the leukocyte populations differed in distribution among the healthy and AUB-affected endometrium, all through the menstruation. Among the differences, CD56+ uNK cells showed the most differences in AUB samples (5). CD56+ uNK cells, CD14+ macrophages, and CD3+ T cells make up the three most dominant leukocyte populations inside the endometrium (Hunt, 1994; Lachapelle et al., 1996). The proliferative endometrium of controls demonstrated 2% of stromal cells to be CD56+ uNK cells, rising to 17% in the late secretory phase. In AUB, CD56+ uNK cells were elevated to 5% in the proliferative phase and to 4% in the early secretory phase but reduced to 10% in the late secretory phase. Thus, in conclusion, the results demonstrated increased uNK cells in the proliferative and early secretory phases, while the same cells were reduced in the late secretory phase in heavy menstrual bleeding [5].

The available modern treatments for abnormal uterine bleeding are satisfactory; however, they have side effects. The ablation of endometrium and levonorgestrel intrauterine systems are effective, safe alternatives, and less invasive than a hysterectomy in females with AUB. A hysterectomy is the perfect AUB treatment, regardless of the suspected cause, when alternate treatments fail. Conventional treatments comprise non-steroidal anti-inflammatory drugs (NSAIDs), oral progesterone, oral progestins, tranexamic acid, and oral, transdermal, intravaginal, and intrauterine hormonal contraception. Herbal therapies for acute intervention comprise herbal anti-inflammatories, herbal coagulants, and astringents. A variety of herbs have been found for treating gynecological diseases, such as irregular menses and heavy menstrual bleeding. Though studies have shown the beneficial effects of herbs on heavy menstrual bleeding, there is no comprehensive study on the effectiveness of herbs used for treating abnormal uterine bleeding [6].

Shalmali, scientifically known as Bombax ceiba, is a traditional medicinal plant widely utilized in various systems of medicine for its therapeutic properties. Found in tropical and subtropical regions, Shalmali is revered for its ability to address a range of health concerns, including uterine bleeding, inflammation, and gastrointestinal disorders. Rich in phytochemicals, Shalmali extract has shown promising results in preclinical studies, demonstrating anti-inflammatory, antioxidant, and anti-hemorrhagic properties. Its efficacy in managing uterine bleeding has been attributed to its ability to modulate gene expression levels associated with hormonal regulation and tissue repair. Moreover, Shalmali extract has been found to exhibit comparable effects to standard pharmaceutical interventions, making it a valuable natural alternative for individuals seeking traditional remedies. Further research is needed to explore its full therapeutic potential and to validate its safety and efficacy in clinical settings.

Gene expression analysis serves as a valuable tool for elucidating the molecular mechanisms underlying the pharmacological effects of herbal extracts. By examining changes in gene expression levels, researchers can gain insights into the biological pathways influenced by these extracts [7]. In the context of gynecological health, gene expression analysis can provide valuable information on the modulation of hormonal signaling, cellular proliferation, and tissue repair processes implicated in uterine bleeding.

Tranexamic acid is a well-established drug commonly used for the management of uterine bleeding due to its antifibrinolytic properties [8]. While effective, tranexamic acid may be associated with certain side effects and contraindications, highlighting the need for alternative treatment options with favorable safety profiles [9]. The comparison of Shalmali extract with tranexamic acid in this study provides valuable insights into its potential as a natural alternative for managing uterine bleeding.

Shalmali is employed therapeutically to address various health issues such as menorrhagia, acute dysentery, and hemoptysis. Additionally, it serves as a regulator in herbal medicine for conditions like menorrhagia, menstrual cramps, and suppressed menses. Thus, the present study aims to provide scientific validation for the treatment of abnormal uterine bleeding. This will be achieved through in vitro investigations of Shalmali extract on key genes associated with uterine bleeding, namely ESR1, CD56, and SDF-1, utilizing a human endometrial stromal cell line (T HESC).

## Materials and methods

The study was conducted at Sree Balaji Medical College and Hospital, Bharath Institute of Higher Education and Research, BIHER, Chennai, India. The chemicals required for the analysis were from SD Fine Chemicals Ltd., solvents from Randox Laboratories, and standards from Sigma Aldrich Company, Inc. The qRT-PCR kits and primers were purchased from Biobasics Inc., Canada.

Test drugs

The resin of Shalmali was obtained from the Indian Medical Practitioner's Cooperative Pharmacy and Stores Ltd., located in Chennai. This resin, marketed under the brand name Shalmali Niryasa, was utilized for this study.

Preparation of Shalmali extract

Dried powder Shalmali resin (15 grams) was weighed in a beaker for sample extraction with the help of ethanolic and hydroethanolic solvents (20% ethanol and 80% distilled water) as solvents. The extraction was done with 100 ml of every solvent for 48 hours. After extraction, the corresponding solutions were filtered and concentrated under abridged pressure, and the extract was kept in the refrigerator at 4°C.

In vitro studies of Shalmali on T HESC cells

Human Endometrial Fibroblast Cell Culture

The T HESCs immortalized cell lines were procured from ATCC-CRL-4003. The cells were cultured in the growth medium in an equal proportion of Dulbecco’s Modified Eagle’s medium and Ham’s F12 medium with 1 mM sodium pyruvate, 3.1 g/L glucose, and depletion of phenol red added with sodium bicarbonate (1.5 g/L), 90% charcoal/dextran treated with 10% fetal bovine serum, 1% ITS, and premix 500 ng/mL puromycin. The cells were kept in a 6% CO2 humidified incubator at 37°C. A thermometer was kept inside an incubator to provide a free readout of the culture temperature.

Gene Expression Analysis of CD56, ESR1 and SDF-1

Total RNA was isolated from treated and untreated cells using the One Step-RNA Reagent from Biobasic Inc. After RNA isolation, the RNA was transcribed using EasyScript Plus^TM^ Reverse Transcriptase, a novel recombinant reverse transcriptase known for its high efficiency in synthesizing first-strand cDNA from RNA templates. For the RT-PCR reaction, 1-2µg of RNA was utilized, as compared to 1-10µl of total RNA isolate as described previously (Singh et al., 2014). Samples were then frozen in liquid nitrogen and stored at −80°C until further use.

For the extraction of the epidermal layer, samples (10 and 5-10 µg) were minced and subjected to total RNA extraction using Trizol® reagent (Invitrogen, USA). The resulting RNA pellet was dissolved in DEPC-treated distilled H2O (10 µL). Reverse transcription was performed with RNA samples confirmed to be free of protein and phenol by UV spectrophotometry. Total RNA (0.5 µg) was mixed with oligo dT (2 µL) and DEPC-treated H2O (14 µL), heated at 65°C for five minutes, then chilled on ice. Subsequently, dNTP 10 mM (2 µL), 4 µL of 10 mM dithiothreitol, and 8 µL of first strand buffer were added, and the solution was incubated at 55°C before the addition of 200 U Superscript II®. The reaction was carried out at 55°C for 60 minutes, followed by 15 minutes at 85°C. The resulting cDNA was stored at -80°C until further processing for PCR.

The obtained cDNA was amplified by PCR, with gene-specific primers used to amplify the CD56, ESR1, and SDF-1 genes. Primer specificity was confirmed by agarose gel electrophoresis and melting profile analysis of the amplicon. Dissociation curve analysis was performed to ensure the specificity of the amplified product. Agarose gel (1.5%) in 1X TAE buffer was prepared, and the PCR product was mixed with loading dye before loading onto the gel along with a 1KB Ladder as a reference. Gel electrophoresis was conducted at 50 V for 90 minutes, and the bands were visualized and captured digitally. Band intensity was analyzed using Gel Pro Analyzer software, version 4.0. The relative expression of CD56 and SDF-1 genes was normalized to the reference gene, β-actin mRNA, while the reference gene GAPDH was used for normalization of ESR1. The list of primer sequences is given in Table 1.

**Table 1:** List of primer sequences in the study

Genes	Forward primer	Reverse Primer
β-actin	5′- TGACGGGGTCACCCACACT -3’	5′-CTTAGAAGCATTGCGGTGG-3′
CD56	5′- CTCCACCCTCACCATCTAT-3’	5′- TCGCCTGTAACCACACACT-3’
SDF-1	5’-CACAGATTCCTTGCCGAG-3’	5’-GGCAAGCAGAGATCAGAA-3’
GAPDH	5′- TCTAGACGGCAGGTCAGGTCC -3’	5′- CCACCCATGGCAAATTCCATG -3′
ESR1	5′- CCGGCTCCGTAAATGCTACG -3’	5′- TCC AGCAGACCCCACTTCAC-3’

## Results

Effect of Shalmali resin extract on ESR1, CD56, and SDF-1 genes

Effect of Shamali Extract on ESR1

**Figure 1:**
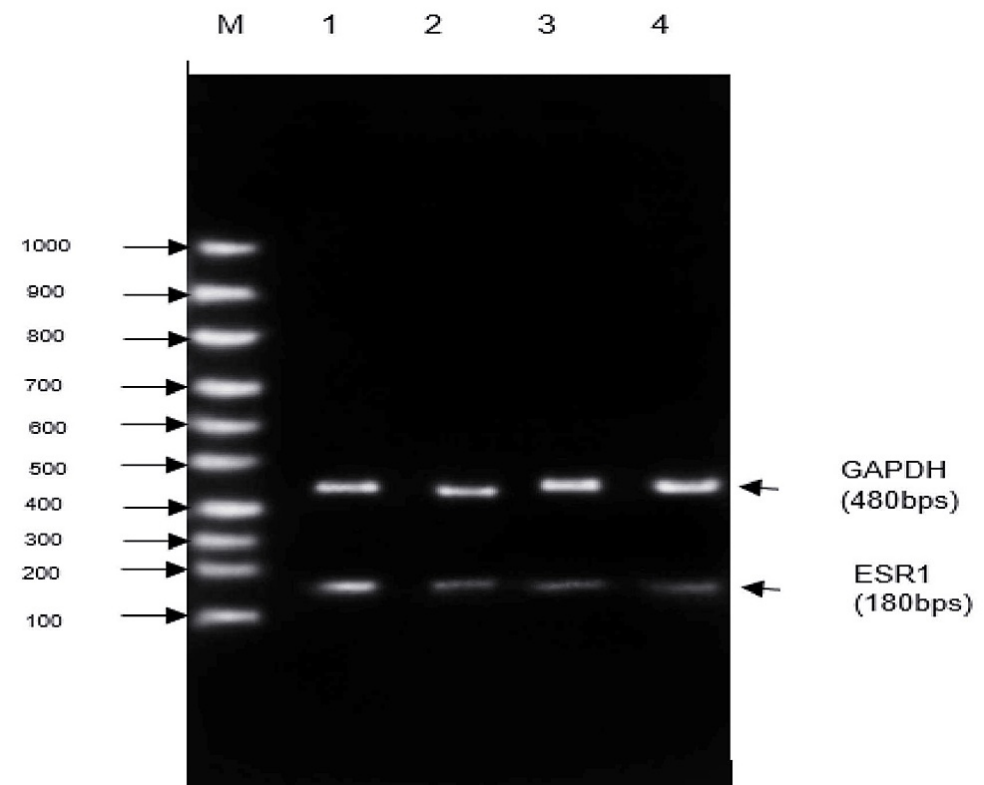
Agarose gel electrophoresis image of ESR1 gene expression in T HESC cells treated with Shalmali extract Lane M: marker lane (100-1000 bp); Lane 1: T HESCs untreated control; Lane 2: T HESCs + SE (100 µg); Lane 3: T HESCs + SE (200 µg); Lane 4: tranexamic acid (10 mg).

**Figure 2:**
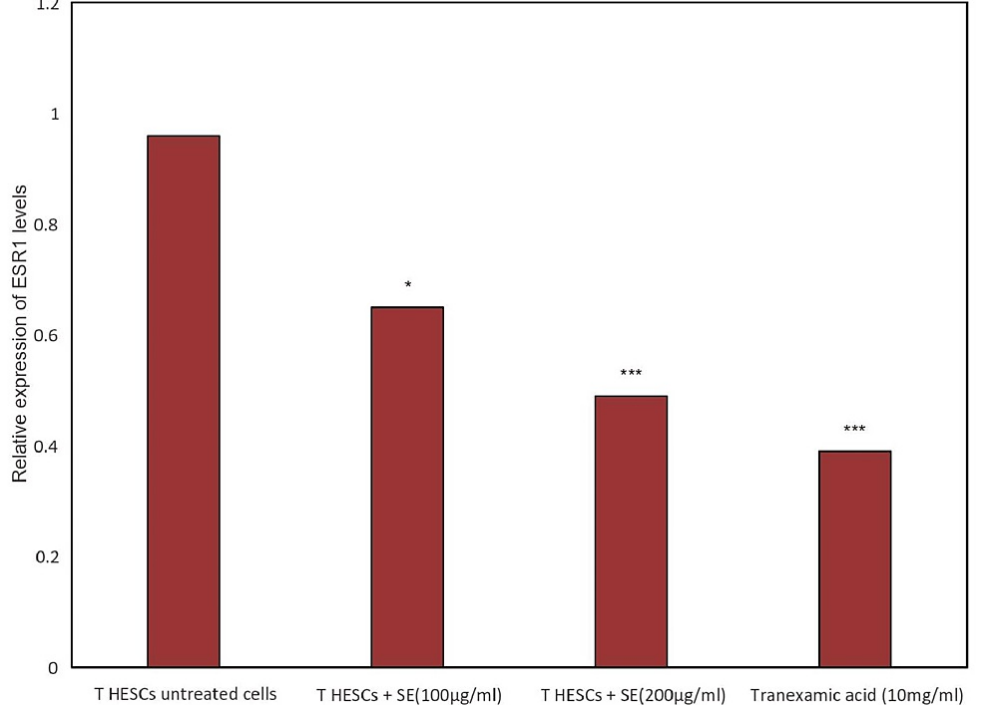
Effect of Shalmali extract (SE) treatment on the expression of ESR1 using GAPDH as an internal control The bar shows the mean ± SEM of three independent experiments. The level of the ESR1 gene was significantly higher in the untreated control group than that of the SE (*p<0.05; ***p<0.01 and ***p<0.001) standard-treated groups. GAPDH: glyceraldehyde-3-phosphate dehydrogenase

The level of the ESR1 gene was significantly higher in the untreated control group than that of the SE (*p<0.05; ***p<0.01 and ***p<0.001) standard-treated group (Figure 1). The result of the study showed that highly increased levels of ESR1 gene expression were seen in the untreated control group of T HESCs. In T HESCs, cells treated with Shalmali extract showed decreased expression based on increasing doses (Figure 1). The standard group of 10 µg/ml tranexamic acid showed reduced gene expression of ESR1 compared with the untreated group. Results of different concentrations of Shalmali extract (100 µg/ml and 200 µg/ml) and the relative expression of ESR1 levels were graphically represented in Figure 1. The agarose gel electrophoresis of ESR1 gene expression shows that gene levels are reduced in T HESC cells treated with Shalmali extract in comparison to the standard drug (Figures 1, 2).

Effect of Shamali Extract on CD56

**Figure 3:**
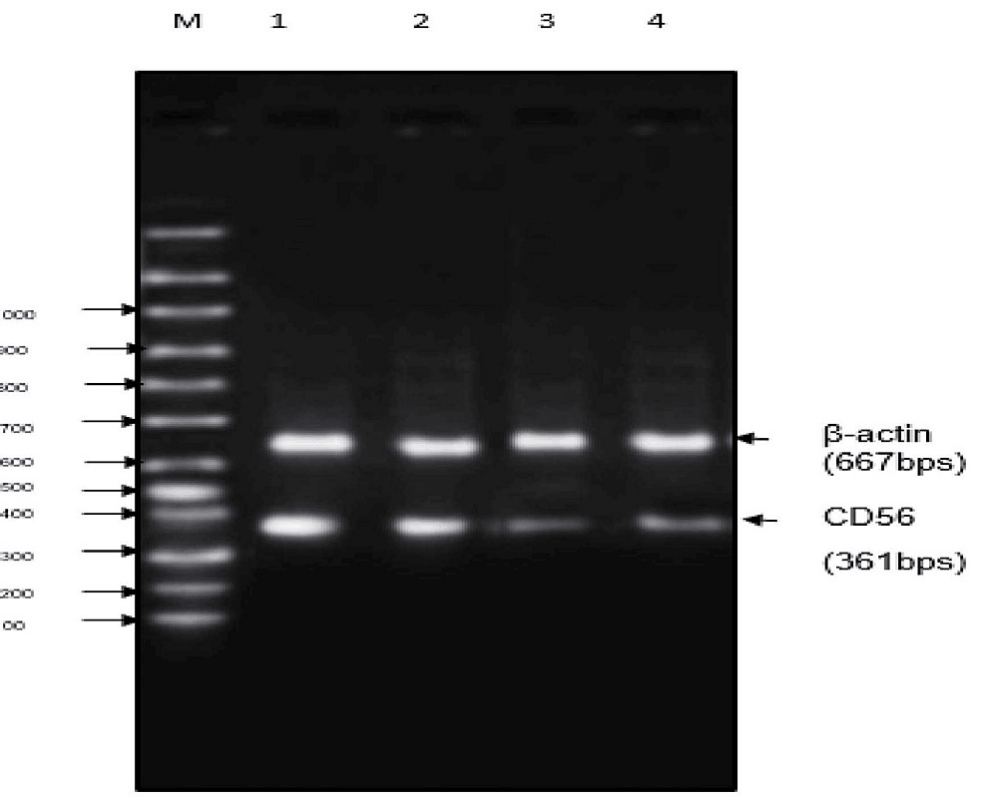
Agarose gel electrophoresis image of CD56 gene expression in T HESC cells treated with Shalmali extract Lane M: marker lane (100-1000 bp); Lane 1: T HESCs untreated control; Lane 2: T HESCs + SE (100 µg); Lane 3: T HESCs + SE (200 µg); Lane 4: tranexamic acid (10 mg).

**Figure 4:**
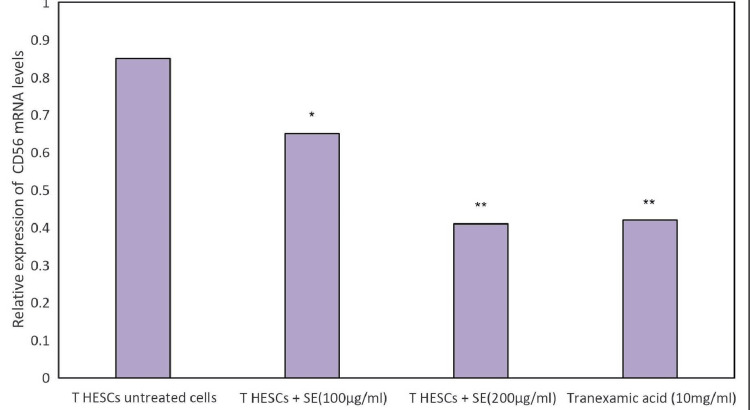
Effect of Shalmali extract (SE) treatment on the expression of the CD56 gene using β-actin as an internal control The bar shows the mean ± SEM of three independent experiments. The level of the CD56 gene was significantly higher in the untreated control group than that of the SE and standard-treated groups.

The level of the CD56 gene was significantly higher in the untreated control group than in the Shalmali extract and standard-treated groups. In T HESCs, cells treated with Shalmali extract showed decreased expression based on increasing doses. The standard group of 10 µg/ml tranexamic acid showed decreased gene expression of CD56 compared with the untreated group. Results of different concentrations of Shalmali extract, including 100 µg/ml and 200 µg/ml, and the relative expression of CD56 levels were graphically represented in Figures 3, 4.

The level of the CD56 gene was significantly higher in the untreated control group than that of the tranexamic acid-treated group. Significant reduction has been found in Shalmali extract treated with T HESC cells (200 µg/ml) concentration close to that of tranexamic acid treatment when compared with the untreated T HESC group. It was observed that the relative expression of CD56 levels decreased with increasing concentrations of test compounds. Agarose gel electrophoresis images of CD56 gene expression show that gene levels are reduced in T HESC cells treated with Shalmali extract as compared to the standard drug (Figures 3, 4).

Effect of Shamali Extract on SDF-1

**Figure 5:**
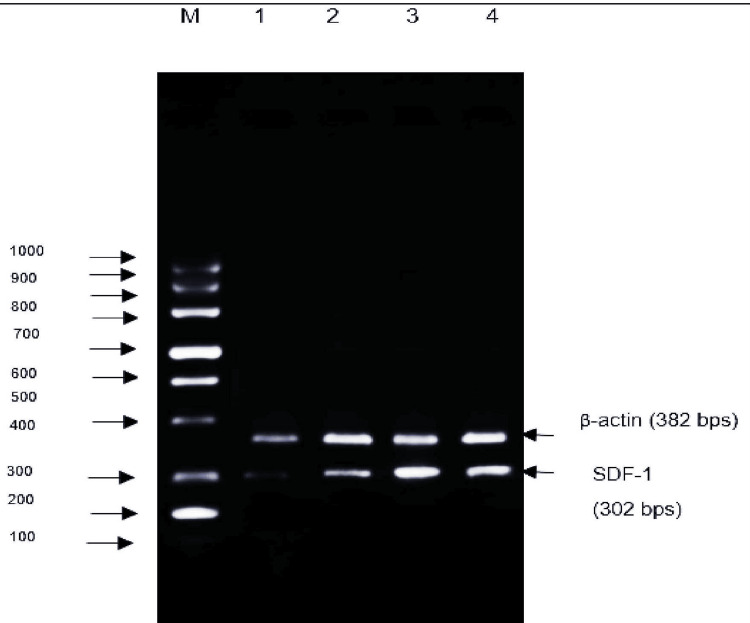
Effect of Shalmali extract treatment on the expression of SDF-1 gene using β-actin as an internal control Lane M: marker lane (100-1000 bp); Lane 1: T HESCs untreated control; Lane 2: T HESCs + SE (100 µg); Lane 3: T HESCs + SE (200 µg); Lane 4: tranexamic acid (10 mg).

**Figure 6:**
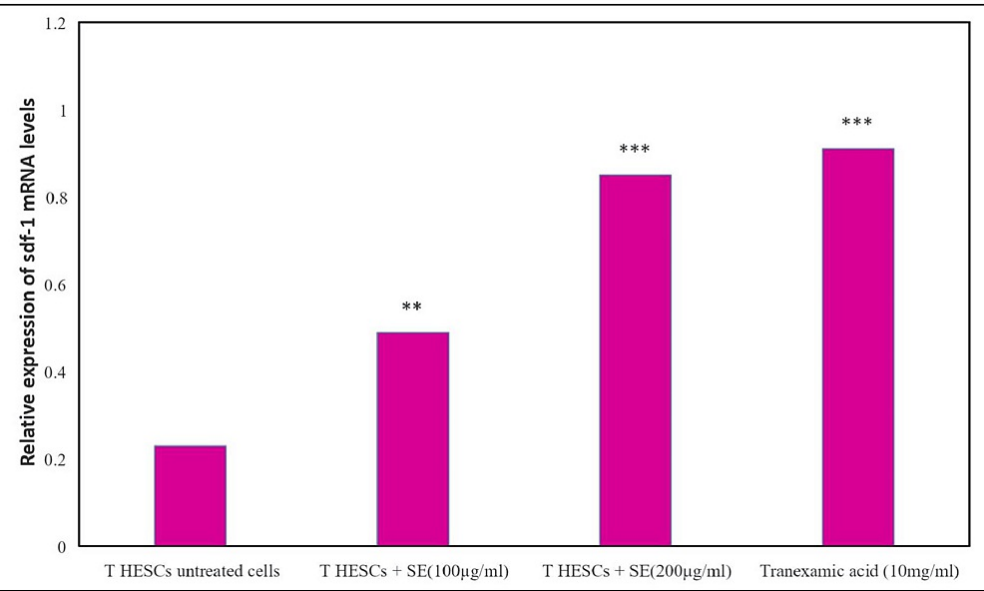
Effect of Shalmali extract (SE) treatment on the expression of the SDF-1 gene using β-actin as an internal control The bar represents the mean ± SEM of three independent experiments. The level of the SDF-1 gene in the untreated control group was significantly different than that of the SE (**p<0.01 and ***p<0.001) and standard-treated groups.

Figure 5 shows a low level of SDF-1 gene expression in the untreated control group of T-HESCs. In T HESCs, cells treated with Shalmali extract showed increased expression based on the increasing dose. The standard drug group also showed increased expression compared with the untreated group. The expression of stromal cell-derived factor was measured using β-actin as an internal control and was observed to be lower in untreated T HESC cells. T HESCs treated with 200 μg/ml showed higher expression of SDF-1 mRNA, similar to tranexamic acid, compared with the untreated T HESCs group in Figure 6. Agarose gel electrophoresis image of SDF-1 gene expression in T HESC cells treated with Shalmali extract also displays the increased expression of SDF-1 like that of standard drug tranexamic acid administration (Figure 6).

## Discussion

The study reveals the effects of Shalmali extract and tranexamic acid on m-RNA gene expression levels, focusing on the ESR1, CD56, and SDF-1 genes in T HESC cells. The results indicate a significant increase in ESR1 gene expression in the untreated control group compared to the standard-treated groups, indicating the potential of tranexamic acid to reduce ESR1 expression. Moreover, Shalmali extract exhibited a dose-dependent decrease in ESR1 expression at 200 µg/mL concentration, showing comparable effects to tranexamic acid. Similarly, CD56 gene expression was elevated in the untreated control group; however, both Shalmali extract and tranexamic acid treatments led to reductions in CD56 expression, with the extract showing promised efficacy at 200 µg/ml concentration. Additionally, SDF-1 gene expression increased in cells treated with Shalmali extract at higher doses, resembling the effects observed with tranexamic acid treatment. Agarose gel electrophoresis further confirmed the gene expression studies, indicating a potential role of Shalmali extract in modulating gene expression associated with T HESC cells. Overall, the results suggest the therapeutic potential of Shalmali extract in influencing gene expression related to endometrial decidualization [10].

Estrogen, acting through its receptors, regulates normal endometrial epithelial and stromal cell proliferation. However, excessive estrogen levels can lead to endometrial tumorigenesis. Contrastingly, progesterone acts as a natural tumor suppressor for the endometrium, counteracting the pro-proliferative effects of estrogen by inhibiting inflammation, arresting the cell cycle, and promoting differentiation and apoptosis of glandular epithelial cells. Any imbalance between progesterone and estrogen, such as prolonged estrogenic stimulation or inadequate progesterone activity, can contribute to excessive endometrial growth and potential tumorigenesis. After menstruation, the endometrial cells, under the influence of estrogen, regenerate from the cells of the remaining stratum basalis, leading to the proliferation and thickening of the endometrial layer. Estrogen, through its receptors ERα (ESR1) and ERβ (ESR2), plays a pivotal role in endometrial cell proliferation, and aberrant estrogen signaling can contribute to endometrial cancer development. This study revealed elevated levels of ESR1 gene expression in untreated T HESC's control group, whereas cells treated with Shalmali extract exhibited dose-dependent decreases in expression. Compared to the untreated group, the standard treatment group showed reduced expression. Cytotoxicity assays at various concentrations of Shalmali extract (100 and 200 μg/ml) demonstrate significant reductions in ESR1 expression, particularly at 200 μg/ml, akin to tranexamic acid treatment. This reduction in gene expression was consistent with increasing concentrations of the test compounds.

In recent years, there has been a growing interest among researchers in investigating the therapeutic potential of herbal remedies for various health conditions, including gynecological disorders [11]. This interest stems from the recognition of the rich pharmacological diversity present in medicinal plants and the potential they hold for providing safe and effective treatment options [12]. Bombax ceiba, commonly known as Shalmali, is a medicinal plant widely used in traditional medicine systems for its therapeutic properties. Previous studies have reported various pharmacological activities associated with Shalmali extract, including anti-inflammatory, analgesic, and wound-healing effects [13]. However, its specific effects on gynecological health, particularly in the management of uterine bleeding, remain relatively unexplored. Traditional medicinal systems, such as Ayurveda, Traditional Chinese Medicine (TCM), and indigenous healing practices, often rely on botanical remedies for addressing gynecological concerns, including uterine bleeding. The utilization of herbal formulations for managing reproductive tract disorders has been deeply ingrained in traditional medicine practices across various cultures for centuries [14]. This study revealed that the level of the CD56 gene, a homophilic binding glycoprotein expressed on the surface of neurons, glia, and skeletal muscle, was significantly higher in the untreated control group than that of the SE and standard-treated groups. Studies showed that in abnormal uterine bleeding, CD56 genes were enhanced in the early secretory and proliferative stages; however, they decreased in the late secretory phase, which signifies CD56 dysregulation in abnormal uterine bleeding (AUB). The functional consequence influences endometrial preparation for menstruation or endometrial vascular development. During the menstrual cycle, there is a significant increase in CD56 gene expression in the human uterus, coinciding with the surge in luteinizing hormone (LH) that initiates final oocyte maturation. While some CD56 genes are present in the uterus before ovulation, their abundance rapidly increases due to the proliferation of resident CD56 lymphocytes and the recruitment of circulating CD56 lymphocytes. This rapid enhancement of CD56 gene expression aligns with the onset of decidualization, a process where uterine stromal cells transform into secretory decidual cells. CD56 cells in the uterus undergo differentiation and proliferation, becoming the predominant lymphocytes in the post-ovulatory uterus.

A study indicated that the chemokine stromal cell-derived factor-1 (CXCL12 or SDF-1) may facilitate the homing of endothelial progenitor cells (EPCs) to the endometrium. Elevated plasma levels of CXCL12 were observed during the proliferative phase, exhibiting a negative correlation with blood EPC-colony forming units (EPC-CFUs). Stromal cells secrete CXCL12, establishing a chemoattractant gradient around blood vessels, which attracts circulating EPCs to integrate into the vessel walls. This mechanism may contribute to the repair of minor damage to vessel walls and could also be activated during events triggering angiogenesis. Evidence across various systems suggests a model where disruption of the CXCL12/CXCR4 axis between bone marrow stroma cells and EPCs leads to the release of EPCs from the bone marrow. Subsequently, blood EPCs are recruited to sites of active angiogenesis by locally expressed adhesion and chemotactic factors. The abnormal cycling of CXCL12 observed in patients with heavy menstrual bleeding (HMB-E) further supports the notion of dysregulated angiogenesis in abnormal uterine bleeding disorders.

Traditional medicinal practices have long utilized various herbs, such as Bombax ceiba [15], for managing reproductive tract issues like uterine or cervical bleeding. This study focused on the role of Shalmali (Bombax ceiba) extract in addressing uterine bleeding, revealing significant insights into its effects on gene expression. Tranexamic acid is a well-established drug commonly used for the management of uterine bleeding due to its antifibrinolytic properties [8]. While effective, tranexamic acid may be associated with certain side effects and contraindications, highlighting the need for alternative treatment options with favorable safety profiles [9]. The comparison of Shalmali extract with tranexamic acid in this study provides valuable insights into its potential as a natural alternative for managing uterine bleeding.

The findings indicated a decrease in ESR1 and CD56 gene expression with increasing doses of Shalmali extract, along with an increase in SDF-1 gene expression. Notably, the extract exhibited comparable effects to the standard drug, tranexamic acid, in reducing gene expression levels. Agarose gel electrophoresis images further supported these findings, showing reduced gene expression in cells treated with Shalmali extract akin to those treated with the standard drug. These results underscore the potential of Shalmali extract as a therapeutic option for managing uterine bleeding, warranting further investigation into its efficacy and safety.

Shalmali has been extensively studied in vitro and in vivo for pharmacological activities, including antioxidant, antimicrobial, anticancer, anti-inflammatory, hypotensive, hypolipidemic, antidiabetic, and analgesic properties; however, all the activities cannot be proven in a single study. The synergism of active components present in Shalmali may be a contributing factor to its medicinal value. Additionally, Shalmali has been used in cases of abnormal uterine bleeding due to its anti-inflammatory activity. The present study involves a culture study with a minimal number of cells. The precise mechanism of active components in in vitro and in vivo studies is to be investigated for its therapeutic potential.

## Conclusions

In conclusion, abnormal uterine bleeding (AUB) significantly impacts the quality of life of affected women and poses considerable challenges to both mental and physical health. Through our study, we have demonstrated the therapeutic potential of Shalmali extract in addressing AUB. By treating T HESC cells with Shalmali extract, we observed beneficial effects on abnormal uterine bleeding disorders, supported by strong evidence from gene expression analysis. These findings provide scientific validation to society, suggesting that Shalmali resin extract could serve as an effective intervention in the management of abnormal uterine bleeding, offering hope for improved outcomes and enhanced well-being for women affected by this condition.
